# Integral Roles of Specific Proteoglycans in Hair Growth and Hair Loss: Mechanisms behind the Bioactivity of Proteoglycan Replacement Therapy with Nourkrin® with Marilex® in Pattern Hair Loss and Telogen Effluvium

**DOI:** 10.1155/2020/8125081

**Published:** 2020-05-05

**Authors:** Jan Wadstein, Erling Thom, Aida Gadzhigoroeva

**Affiliations:** ^1^Research and Development, Wadlund A/S, Lund, Sweden; ^2^ETC Research and Development, Oslo, Norway; ^3^Moscow Scientific and Practical Centre of Clinical and Aesthetic Dermatology and Venerology, Moscow City Health Department, Moscow, Russia

## Abstract

Follicular proteoglycans are key players with structural, functional, and regulatory roles in the growth and cycling behaviour of the hair follicles. The expression pattern of specific proteoglycans is strongly correlated with follicular phase transitions, which further affirms their functional involvement. Research shows that bioactive proteoglycans, e.g., versican and decorin, can actively trigger follicular phase shift by their anagen-inducing, anagen-maintaining, and immunoregulatory properties. This emerging insight has led to the recognition of “dysregulated proteoglycan metabolism” as a plausible causal or mediating pathology in hair growth disorders in both men and women. In support of this, declined expression of proteoglycans has been reported in cases of anagen shortening and follicular miniaturisation. To facilitate scientific communication, we propose designating this pathology “follicular hypoglycania (FHG),” which results from an impaired ability of follicular cells to replenish and maintain a minimum relative concentration of key proteoglycans during anagen. Lasting FHG may advance to structural decay, called proteoglycan follicular atrophy (PFA). This process is suggested to be an integral pathogenetic factor in pattern hair loss (PHL) and telogen effluvium (TE). To address FHG and PFA, a proteoglycan replacement therapy (PRT) program using oral administration of a marine-derived extract (Nourkrin® with Marilex®, produced by Pharma Medico Aps, Aarhus, Denmark) containing specific proteoglycans has been developed. In clinical studies, this treatment significantly reduced hair fall, promoted hair growth, and improved quality of life in patients with male- and female-pattern hair loss. Accordingly, PRT (using Nourkrin® with Marilex®) can be recommended as an add-on treatment or monotherapy in patients with PHL and TE.

## 1. Introduction

Proteoglycans are structural and functional macromolecules consisting of a core protein covalently attached to one or more glycosaminoglycan chains via O-(serine/threonine) or N-(asparagine) linkages. Glycosaminoglycans, or mucopolysaccharides, are long unbranched polysaccharides comprising a repeating disaccharide unit. Five types of glycosaminoglycans, heparan sulphate (HS), chondroitin sulphate (CS), dermatan sulphate (DS), hyaluronan (HA), and keratan sulphate (KS), in combination with a limited number of core proteins constitute a wide variety of proteoglycans. Although first identified as extracellular matrix (ECM) components, these macromolecules are abundant in intracellular, cell membrane and pericellular (basement membrane zone) matrices. Proteoglycans are generally categorised based on their cellular/subcellular distribution and structural features [1]. Determined by its function, each class of proteoglycans exhibits distinctive cellular and tissue distribution patterns [2].

Decades of rigorous research have unveiled that, in addition to their pivotal structural roles, proteoglycans are physiologically active molecules that regulate cellular processes. These regulatory roles are partly implemented through binding of proteoglycans to receptor tyrosine kinases and modifying a range of downstream signalling pathways [3]. Modulated metabolic pathways by proteoglycans govern cell growth, adhesion and migration, collagen fibrillogenesis, immune functions, and tissue repair [2]. Their omnipresence and diverse functionality imply that any structural distortion or imbalance in the production and degradation of proteoglycans may lead to disorder. Typical examples are mucopolysaccharidosis, tumorigenesis, atherosclerotic plaque formation [4], osteoarthritis [5], and hair growth disorders [6].

The current review aims to present an up-to-date account on the roles of specific proteoglycans in the life cycle of the hair follicle and discuss their relevance to the pathophysiology of different types of hair loss. A concise discussion on the theoretical and experimental basis for the development and implementation of a novel class of hair loss therapeutics, that is, proteoglycan-based therapies, is provided as well. As described, available clinical evidence for the efficacy of this emerging therapy, currently known as oral proteoglycan replacement therapy (PRT) using Nourkrin® with Marilex® (produced by Pharma Medico Aps, Aarhus, Denmark), has been promising in various types of hair loss in both men and women.

## 2. The Presence of Proteoglycans Inside the Hair Follicle

The hair follicle is a product of an intricate mesenchymal-epithelial interaction and the only mammalian organ that undergoes recurring fibre production (anagen), degradation (catagen), resting (telogen), and regeneration (neogen) cycles [7]. Maintaining such a dynamic system demands a finely regulated and timely regulated equilibrium between a multitude of dermal and epidermal components including cellular and extracellular elements.

A conspicuous association between fluctuations in follicular glycosaminoglycans and phase shifts during a hair growth cycle was originally reported in the late 60s [8] and was later described in detail in rats [9] and humans [10]. Since then, decades of research [9–15] have evidenced that proteoglycans and their glycan moieties exert integral functions in the development and growth regulation of hair follicles. Histological studies have revealed that hair follicules express a distinctive composition of proteoglycans that contrasts with their surrounding dermal environment [16]. Interestingly, the distribution of these specialised proteoglycans undergoes dramatic alterations throughout a hair growth cycle [15, 17]. This dynamic expression pattern and its plausible functional relevance are described in this section.

Overall, it appears that a model for hair growth physiology/pathophysiology without taking the role of proteoglycans into consideration would be rather inadequate and incomplete.

### 2.1. Follicular Distribution of Proteoglycans

Distribution patterns of proteoglycans in and around the hair follicles have been specified using immunohistochemistry and immunofluorescence techniques. Sharp differences in the composition of ECM between perifollicular regions and surrounding dermal and epidermal zones have been demonstrated by replicated assays [7, 11]. This distinct configuration has provided important clues for understanding the roles of each proteoglycan in the function of the hair follicle miniorgan.

In an anagenic hair follicle, the following classes of proteoglycans are expressed [18]:

1. Lecticans or hyalectans: this family of proteoglycans comprises four members, of which two are found in the mesenchymal parts of the hair follicle. Versican, a large extracellular CS proteoglycan, is highly expressed in the dermal papilla (DP) and the proximal parts of the connective tissue sheath (CTS), while the presence of aggrecan is restricted to the distal parts of the CTS and under the hair matrix [7, 16]. The tridomain structure of these proteoglycans allows them to simultaneously bind to lectins, hyaluronans, and other glycosaminoglycans and function as molecular bridges between the cell surface and ECM [19].
2. Small leucine-rich proteoglycans: of this family, decorin and biglycan are measurably expressed in different follicular regions. Decorin has been identified in the ECM of the DP and follicular bulge [13, 20], whereas biglycan is strongly expressed in the basement membrane and CTS along the hair follicle [16].
3. Basement membrane proteoglycans: HS proteoglycans, perlecan and CD44, and CS proteoglycan, bamacan, are present in the follicular basement membrane [7, 16].
4. Membrane-associated HS proteoglycans: Syndecan-1 is specific to the epithelial parts of the hair follicle and has been spotted in the outer and, to a lesser extent, inner root sheaths and the hair shaft [16].

This typical anagen distribution is known to undergo extensive transformations during a normal hair growth cycle.

### 2.2. Redistribution during the Hair Growth Cycle

As major components of follicular architecture, proteoglycans are part of the periodic size and morphology transformations of the hair follicle [10, 21]. Evidence shows that the redistribution pattern of follicular proteoglycans follows a proactive, rather than passive, pattern, disproportionate to the relative changes in the size of different follicular regions [15, 16]. This pattern supports the functional involvement of specific proteoglycans in follicular growth cycle. Especially that the proteoglycan-rich mesenchymal parts, DP and CTS, display substantial cycle-associated plasticity [22]. Human studies have shown that a significant increase in the volume of the DP from telogen to anagen phase is due to variations in both the number of cells and the amount of ECM per cell [23]. During telogen-to-anagen transition, in parallel with doubling the number of DP fibroblasts, the volume of proteoglycan-rich ECM increases to support the development and survival of the cells [10]. Therefore, keeping the “per-cell ECM volume” within a normal range is crucial. Knowing that proteoglycans of the ECM are highly bioactive, a relatively small change in their per-cell amount is likely to exert disproportionately large functional effects [23].

Extensive studies over decades have unveiled the molecular details of the cyclic redistribution of follicular proteoglycans. It has been reported that CS proteoglycans start to fade from the DP in early catagen and later from the CTS in mid-catagen, leaving close to none staining at telogen. Notably, these changes are merely observed in the lower parts of the follicle where cell proliferation is active [10]. The expression of versican and decorin particularly displays a complete correlation with the growth activity of the hair follicle as they reach their peak concentration in DP and CTS at the height of anagen IV. In support of this, gene expression analyses have evidenced a more than 5× increase in decorin mRNA during middle-to-late anagen. As the follicle enters catagen, decorin expression retreats to the base of the papilla and neck regions (where stem cell populations reside) and entirely disappears by the end of telogen. Later, concomitant with the restoration of hair production in anagen II, CS proteoglycans start to reaccumulate to foster a new cycle [12, 13, 24]. By contrast, HS proteoglycans, concentrated in epithelial parts of the hair follicle, show little variation during anagen-to-telogen transitions and vice versa [10].

**Figure 1:**
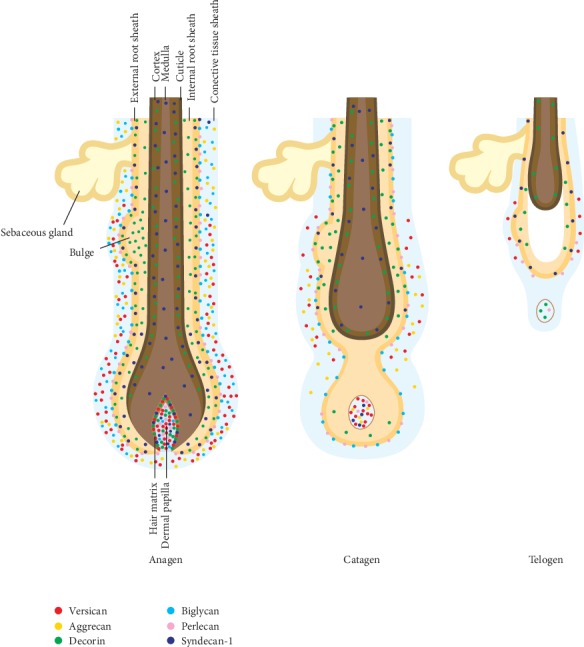
Redistribution of key follicular proteoglycans during the hair growth cycle, which supports their functional roles [12, 16, 21, 24, 25].

Tracing the expression of glycosaminoglycans in different phases of the hair cycle has produced similar patterns of expression and degradation. Total glycosaminoglycans as well as HA and CS reach their peak at mid-anagen and start to decline when the follicle enters catagen [8].  Figure 1 illustrates the cyclic redistribution of key proteoglycans during a normal hair growth cycle.

## 3. Functional Roles of Proteoglycans in the Hair Growth Cycle

After its embryonic formation, the hair follicle shifts periodically between two distinct states, active and dormant; hence, it is referred to as a “bistable miniorgan” [26]. This behaviour, which entails cyclic structural changes and activation of follicular stem cells, relies on a delicate homeostatic balance between a multitude of signalling pathways, receptors, growth modulators, transcription factors, circadian molecular clock genes, cytokines, neuropeptides, hormones, and prostaglandins [18]. When an imbalance in this multiparametric equilibrium exceeds a certain threshold, a leap from one state to another will be triggered [7, 26]. This model predicts that by altering enough parameters, any hair follicle can be induced to enter or remain in one of the two steady states. Telogen effluvium (TE) and pattern hair loss (PHL) are clear manifestations of this prediction (elaborated in later sections).

As the mitotic activity of bulbar epithelium correlates with the volume of the DP [27], a special focus has been placed on the role of the DP in controlling the growth and morphogenesis of human hair follicle [28]. It appears that a reciprocal and interdependent dialogue between the proteoglycan-rich ECM and morphogen-producing fibroblasts within the DP governs the dynamics of follicular phase shift [29]. Proteoglycans are the major component of the ECM microenvironment with direct and indirect cell signalling contributions [30] and independent roles in regulating the hair follicle growth status. Distinct clues for the vital functional and regulatory roles of proteoglycans in the hair follicle originate from the following observations:

1. The expression and redistribution patterns of follicular proteoglycans are proactively correlated with the accumulation and activity of fibroblasts and stem cells [12] (Section 2.2).
2. A pathological increase in deposition of proteoglycans and their moieties in connective tissues, i.e., in mucopolysaccharidoses and myxoedema, leads to hypertrichosis, and the scalp hair becomes extremely coarse and straight [31–33].
3. Certain types of proteoglycans act directly as potent anagen inducers to the extent that their local injection leads to hair growth induction and prolongation [13, 24].
4. Gene silencing of a specific proteoglycan, versican, results in the loss of aggregative growth in DP cells and a significant decline in their proliferative ability [34], whereas its forced expression restores the hair inducibility of DP cells [14].

It is thus not surprising to learn that specific proteoglycans are necessary for the development and maintenance of a healthy anagen phase and can act as potent phase-shifting agents in vivo (see below). This review discusses the diverse functional roles of proteoglycans in the hair follicle under three categories:

### 3.1. Anagen Induction

Anagen is a period of organ regeneration triggered by the activation of various growth factors, neural mediators, and metabolic pathways to which the epithelial-mesenchymal interaction is central [35]. This phase entails a complete regeneration of the lower portion of the follicle and proliferation and differentiation of keratinocytes to generate a new hair shaft. Evidently, the fibroblasts of the DP initiate anagen by secreting anagen inducers, such as Wnt, sonic hedgehog, bone morphogenetic proteins, and fibroblast growth factors (FGFs) [14]; however, this activity is strictly reliant on the presence of a proteoglycan-rich microenvironment and will quickly be lost if DP cells are separated from their ECM [36].

Because of having multiple active domains [1], proteoglycans can regulate the activation of growth factors and other anagen inducers [7, 30]; hence, their concentration is a determining factor for the interaction cascades that lead to anagen initiation. For instance, lecticans contain epidermal growth factor (EGF)-like repeats on the COOH-terminal side of the core protein and perform EGF-like activities [1]. This is important because the EGF receptor signalling is necessary for the initiation of hair follicle cycling [37]. The anagen-inducing effect of versican has been confirmed by in vivo experiments showing that the expression level of versican is significantly correlated with the hair-inductive ability of DP cells [14]. In addition, the induced expression of versican can restore the hair inductivity of inactive mesenchymal cells [18]. As an active macromolecule, versican is highly interactive and appears to induce anagen via its G1 domain. Research shows that this domain stimulates cell proliferation by destabilising cell adhesion and, probably, binding to the cell-surface proteins with signal transmission roles, e.g., CD44 and integrin-*β*1 [4]. In effect, the key growth-inducing activity of Wnt/*β*-catenin pathway is mediated by its regulatory effects on the expression of versican in DP cells [38].

Another key proteoglycan with anagen-inducing properties is decorin [13]. This small extracellular macromolecule acts as a signalling component through the canonical insulin-like growth factor (IGF) signalling cascade and directly regulates cell death and synthesis of other matrix constituents [39]. Decorin actively blocks transforming growth factor beta 1 (TGF-*β*1), a potent apoptosis and catagen inducer [40], which makes it a potential anagen inducer. This mechanistic extrapolation has been experimentally verified by interventional studies on C57BL/6 mice. Remarkably, administration of decorin during telogen had caused a premature onset of anagen [24].

Bioactive proteoglycans that influence the hair follicle's telogen-to-anagen transition are not limited to lecticans and decorin. There is evidence to substantiate that membrane-associated HS proteoglycans serve as low-affinity receptors to a number of growth factors, such as FGFs, and thereby act as reservoirs and presenters of growth factors to their high-affinity receptors. Furthermore, HS proteoglycans are actively involved in the activation of signalling receptors via contributing to their dimerisation [41]. Particularly, syndecans encourage proangiogenic signalling by binding to the FGF-2 and vascular endothelial growth factor and presenting them to their receptors and also protecting them from deactivation [4, 11]. Syndecans are as well involved in both noncanonical and canonical Wnt signalling pathway, which is essential for hair formation [42]. Perlecan, a basement membrane HS proteoglycan, expresses bioactivity by promoting mitogenesis and angiogenesis via activation of FGF-2 and FGF-7 receptors [4, 18]. Both HS and CS chains are known to bind to and modulate the functions of a large number of biomolecules important for cellular differentiation and proliferation, including various FGFs, Wnt proteins, sonic hedgehog, bone morphogenetic proteins, and vascular endothelial growth factor [16]. This bioactivity profile underlies the ability of HS and CS glycosaminoglycans to initiate a hair growth cycle when topically administered to lab animals [43].

### 3.2. Anagen Maintenance

During anagen, mesenchymal components of the hair follicle expand dramatically, and the tight cluster of telogen DP cells spreads into a loose ECM pool [44]. Proteoglycans are central to this phenomenon due to their strong hydrophilic nature that creates a loose and hydrated milieu necessary to support cell migration and intercellular interactions. Beyond this, proteoglycans enhance collagen fibril stability and protect fibrils from proteolytic cleavage [45], thus are key modulators of fibrillogenesis. Versican has been shown to enhance the expression of fibronectin and *β*1-integrin that facilitates cell-to-ECM adhesion [46]. A continuous versican gene expression throughout the anagen phase is thus essential for the maintenance of normal hair growth [15, 47]. In turn, another key proteoglycan, decorin, binds to several collagen types and fibronectin to contribute to the lateral growth of fibrils and maintain the ECM integrity [48]. Critical roles of decorin in hair and skin health are directly supported by interventional in vivo experiments [24] and studies on decorin-deficient mice [49]. An analogue of decorin, biglycan, is proven to act as a reservoir for Wnt protein and regulate the Wnt/*β*-catenin pathway, a vital cascade in hair follicle differentiation and cycling [50].

From another perspective, the antiapoptotic properties of specific proteoglycans may be of importance in maintaining the follicular anabolic homeostasis typical of anagen. In particular, decorin inhibits cell apoptosis by enhancing the phosphorylation of Akt via binding to the IGF receptors as well as suppressing the TGF-*β*-dependent apoptosis [39, 48]. This bioactivity may explain the potent in vivo effect of decorin in inducing significant expansions in bulbar volume of hair follicles and delaying catagen progression. This has been demonstrated in vivo, where only 40% of decorin-treated hair follicles entered catagen compared with 80% of the controls [24].

The newly discovered involvement of certain proteoglycans in cellular autophagy presents yet another mechanism of their regulatory function in replicating follicular cells. Both decorin and perlecan have been shown to influence autophagy, the former being an inducer and the latter an inhibitor [51]. Due to the fact that hair matrix keratinocytes have more prominent autophagic activity during the proliferative stages of the hair cycle, this bioactivity is deemed relevant for maintaining rapid hair growth during anagen. As evidenced by Parodi et al., enhancing cellular autophagy by intervention corresponds with significant pro-anagen efficacy [52].

Besides their diverse functions in lower parts of the follicle, proteoglycans are vital for the normal function and survival of follicular stem cells. The unique ECM in the bulge/isthmus regions, where the hair stem cells are located, contains specific growth factor receptors and high amounts of versican [12]. Versican expression follows the distribution of germ cells throughout the hair cycle and provides a suitable ECM with enhanced motility. This unique ECM regulates cell migration and proliferation by binding to various components such as HA, type I collagen, tenascin-R, fibulin-1 and -2, fibrillin-1, fibronectin, P- and L-selectin, and chemokines [4]. Besides versican, HS proteoglycans, including syndecans, provide essential support for keeping the pluripotency of human stem cells by acting as signalling cofactors of FGF-2, a growth factor with mitogenic and cell survival activities [47, 53].

### 3.3. Immune Response Modification

It has long been known that the proximal section of anagen hair is an immunologically privileged site [54]. Based on the fact that concurrent with each degenerative phase of the hair cycle, this “hairy privilege” is lost [55], follicular cycling behaviour may, in nature, be an immune response-controlled phenomenon. Hence, intact immune privilege is crucially important for inducing and maintaining the anagen phase, and disruptions in its integrity can lead to disorder, as seen in alopecia areata [56]. Although the full details of its molecular mechanisms are not fully understood, the absence of class-*ΙΙ* major histocompatibility complex (MHC) antigens, Langerhans cells, and natural killers is deemed pertinent to this privilege. Moreover, a profusive presence of proteoglycans in and around the follicular epithelium and DP at anagen is considered an indispensable element that safeguards sensitive follicular elements from immune recognition.

This immunoprotective function of proteoglycans may be exerted by providing a barrier that hinders immune cell trafficking [10, 57]. Perlecan is proposed to function as a “border patrol,” concentrated in the margins that separate different regions of complex tissues. This exceptionally large proteoglycan serves as an extracellular scaffold with five functional domains that interact with a variety of proteins and molecules [58]. In early catagen, marginalisation and disappearance of the protective proteoglycan layer are associated with reappearance of class-*ΙΙ* MHC antigens and a striking migration of macrophages towards the follicle and inside the papilla [10]. This phenomenon suggests a direct involvement of proteoglycans in the modification of immune response during anagen.

Nonetheless, it is noteworthy that proteoglycans possess a complex dual bioactivity in modifying the immune response. This means that under tissue stress and injury, some proteoglycans, such as biglycan, act as ligands and signalling molecules of Toll-like receptors [59], whereas in normal conditions, their presence is essential for controlling inflammatory reactions. More recent studies have brought some of the anti-inflammatory activities of biglycan into light, including its role in regulating the balance of IL-1*β* synthesis [60]. The sum of evidence suggests that biglycan is a switcher between inflammation and autophagy, which determines the chronicity or resolution of an inflammatory response (discussed in detail by Roedig et al.) [61]. This dual pro- and anti-inflammatory character has also been observed in decorin. Although decorin can stimulate inflammatory activity [62], in knock-out models, its expression can limit the infiltration of mononuclear cells and expression of TGF-*β*1 that leads to observable antifibrotic effects [63]. Furthermore, the ability of decorin and biglycan to regulate cell-dependent adhesion and migration further upholds their complex immunomodulatory roles [48].

## 4. Altered Dynamics of Proteoglycan Metabolism in Hair Growth Disorders

Being an intricate system controlled by a multitude of signalling cascades, the hair growth cycle is innately vulnerable to instability due to internal and external influences. The resulting disrupted hair cycle appears to be central to the majority of hair growth disorders including PHL (or androgenetic alopecia) and TE [64]. Considering the integral functions of proteoglycans in the development and growth regulation of hair follicles, it is safe to assume their direct or indirect involvement in the pathogenesis of common hair growth disorders. Admittedly, it is not unprecedented in clinical medicine to recognise the role of proteoglycan dysmetabolism in the pathogenesis of human disorders as in the case of mucopolysaccharidoses [65] and osteoarthritis [66], among others. Pathological implication of proteoglycans even extends to neoplastic disorders, where they purportedly regulate the signalling, adhesion, survival, and angiogenesis of cancer cells [4]. In this context, it is startling why this important pathophysiological aspect of hair growth has not yet received the recognition it deserves.

### 4.1. Follicular Proteoglycans and Pattern Hair Loss (PHL)

PHL is the most common type of progressive hair loss with distinctive presentations in men and women. The current theory on the pathogenesis of PHL holds that an increased level of 5-*α* reductase in PHL-prone hair follicles converts testosterone to dihydrotestosterone (DHT). This potent androgen provokes shortening of the anagen phase and a stepwise miniaturisation of hair follicles via an extraordinarily high number of androgen receptors [67]. While this theory best explains the presentations of male-pattern hair loss (MPHL), the pathophysiology of female-pattern hair loss (FPHL) is still not convincingly understood. Few would disagree that the influence of androgens cannot be the sole aetiology of FPHL due to the fact that PHL has been reported in individuals with the complete androgen insensitivity syndrome [68]. Reasonably, the implication of DHT is less significant in FPHL since women's frontal hair follicles have 40% less androgen receptors, up to 350% less 5-*α* reductase expression, and 600% higher aromatase content than men's [69]. Investigators have accordingly speculated certain roles for hormones other than DHT, such as 17*β*-estradiol and prolactin, in the pathogenesis of FPHL [70].

What both FPHL and MPHL are likely to share is considerable disruption of key downstream factors and signalling pathways that act as the effectors of sex hormone receptors [71]. Prime examples are Wnt/*β*-catenin [72, 73], IGF-1 [74], TGF-*β* [75], and interleukin 6 (IL-6) [76] signalling pathways. Since proteoglycans are known to interact with and modify a number of these pathways [18], their altered synthesis, sulfation, and/or degradation can potentially be a common pathology that adversely influences the growth of hair follicles downstream to the androgen receptor in both men and women. A good example is the Wnt/*β*-catenin signalling pathway that is blocked by the activation of androgen receptors [72, 73] while augmented by certain proteoglycans [42, 50]. In vivo studies have elaborated the involvement of proteoglycans in the release and function of Wingless protein, a homologue of Wnt-1, such that enzymatic digestion of sulphated proteoglycans resulted in significant loss of Wingless protein activity [77].

The fact that DHT mainly targets the proteoglycan-rich DP is a valid clue that points to the disequilibrium of proteoglycans in DHT-affected follicles, while the population of bulge stem cells frequently remains intact [28]. The “DP-centric theory of PHL” is further supported by the experiments showing that susceptible DP cells express an exceeding level of androgen receptors and 5-*α* reductase, while steroid-metabolising enzymes (e.g., 17*β*-hydroxysteroid dehydrogenase) are conspicuously underexpressed in these cells [23, 78]. Follicular miniaturisation in PHL is also suspected to be triggered by alterations within the DP since the size of hair bulb is largely determined by the volume of its DP [23, 79].

As stated earlier, proteoglycans form a specialised milieu around fibroblasts that regulates and governs almost all of the autocrine and paracrine signals being sent to the epithelial precursor cells; hence, their disrupted metabolism, especially within the DP, will affect the normal cycling behaviour of the hair follicle. It hence appears that a certain relative concentration of follicular proteoglycans is necessary for sustaining a normal anagen duration and preventing immature initiation of catagen. Evidence implies that even the slightest alteration in the concentration or composition of follicular proteoglycans can cause disproportionately large functional effects [23]. This can be observed at the end of anagen, when a shrinkage in the proteoglycan-rich, papillary ECM is the first morphological sign of catagen initiation [80]. Thus, we propose that a possible mediator of sex hormone-induced DP dysfunction in PHL may ostensibly be a decline in the ability of cells to synthesise proteoglycans. The suppressive action of androgens on the ability of papillary fibroblasts to synthesise ECM components and its role in the pathogenesis of PHL have previously been discovered through in vitro experiments [81, 82]. This defect undermines the capacity of cells to replenish and maintain a minimum functional concentration of key proteoglycans during telogen-to-anagen transition and throughout anagen. The resulting suboptimal functional state can be referred to as a “state of follicular hypoglycania (FHG).” Premature senescence of DP cells in PHL [83] may also contribute to this phenomenon.

The newly recognised pathophysiological role of FHG and the ability of targeted treatments to address it have recently been outlined in an informative review by Sonthalia et al. [6]. Of note is that FHG in PHL has particularly been evidenced using versican as an archetypal bioactive proteoglycan. Both the gene and protein expression of versican were reduced at anagen in the DP of PHL-affected hair follicles. This underexpression was suggested to be regulated at a transcriptional level since versican mRNA was not detectable in the DP of vellus-like hair follicles. This observation implies that potent androgen-responsive elements may be located in the regulatory sequence of the versican gene [15].

An initial stage of FHG can cause follicular hypotrophy without visibly affecting the size of the follicle. Even this may trigger a negative feedback loop of cellular dysfunction and reduced proteoglycan synthesis in consecutive cycles, resulting in progressively lower per-cell proteoglycan concentration and shorter anagen phases. If this vicious cycle continues for an adequately long time, macroscopic FHG at anagen ensues. Macroscopic FHG underlies visible follicular miniaturisation, referred to as proteoglycan follicular atrophy (PFA), to highlight the significance of proteoglycans in its aetiology. The pathological processes that lead to PFA are expected to occur primarily in the DP at early anagen or early catagen when the DP is exposed to external effectors [84].

**Figure 2:**
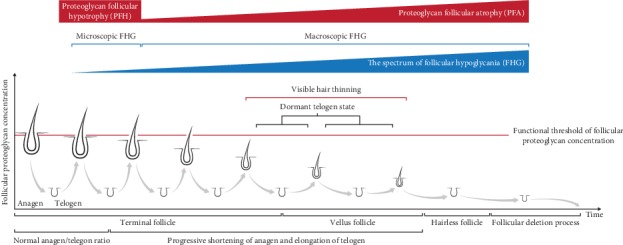
This illustration demonstrates the functional effects of dysregulated turnover of follicular proteoglycans on the growth and cycling behaviour of the hair follicles. As the ability of follicular cells to synthesise proteoglycans diminishes, the anagenic concentration of bioactive proteoglycans falls below a certain threshold leading to progressive degrees of follicular hypoglycania (FHG). Advanced FHG can result in a gradual increase in telogen to anagen duration ratios and decline in the maximum size of the hair follicles. At its early stages, FHG underlies subclinical alterations in the size of the follicle, called proteoglycan follicular hypotrophia (PFH), which advances to more significant stages, referred to as proteoglycan follicular atrophy (PFA). PFA is presented clinically as progressive hair miniaturisation.

PFA may well be the missing mechanism responsible for follicular miniaturisation suggested by the iconic experiment by Hamilton et al. Their findings pointed to the involvement of mechanisms beyond the androgen receptor since androgen withdrawal did not lead to the reversal of hair thinning in PHL [85]. The complete spectra of FHG and PFA and their association with the dysregulation of hair growth cycle and follicular miniaturisation over time are depicted in Figure 2.

A low concentration of follicular proteoglycans may have more far-reaching consequences than just the lack of their structural and functional roles. As explained earlier, proteoglycans and their glycan moieties play significant roles in restoring and sustaining the follicular immune privilege [10, 57]. FHG may thus at least partly explain the perifollicular inflammation frequently implicated in the aetiology of PHL. Human studies have shown that, in a staggering 71% of patients with PHL, histological evidence of perifollicular micro-inflammation and fibrosis, mostly around the miniaturising follicles, can be found [86, 87], and such a finding correlates with poor treatment response [88]. This association highlights another viable mechanism for the involvement of proteoglycans in the pathophysiology of PHL and introduces a potential target for intervention. Overall, regardless of the initial aetiology, FHG is likely to be a downstream phenomenon that underlies the main aspects of PHL in both men and women, which are anagen shortening and follicular miniaturisation.

### 4.2. Follicular Proteoglycans and Telogen Effluvium (TE)

TE is a classic example of increased hair loss secondary to aberrations in the hair growth cycle and shedding of club hairs (exogen phase). Irrespective of the initial trigger, all types of noninflammatory TE have one histologic feature in common, that is, an increased telogen/anagen ratio in scalp follicles [89, 90]. While TE is less likely than PHL to cause long-term hair loss, it usually provokes anxiety and distress disproportionate to its actual severity and prognosis [91]. Hence, providing medical and psychological support is of clinical importance, particularly in the stress-susceptible women suffering from postpartum TE, or telogen gravidarum. This common form of TE is triggered by an abrupt drop in the plasma level of placental oestrogens after childbirth. This hormonal fluctuation invokes a synchronised termination of the anagen phase in a considerable number of scalp hair follicles resulting in an abnormal surge in hair shedding a few months after delivery [90].

Currently, there is some evidence supporting that dysmetabolism of follicular proteoglycans may be implicated in this phenomenon. Human experiments have evidenced that radical hormonal fluctuations during and after pregnancy can significantly affect the homeostasis of tissue proteoglycans. For instance, cervical concentration of versican, decorin, and HS proteoglycans undergo dramatic alterations during gestation and delivery [92, 93]. Arguably, a change of much lower magnitude in proteoglycan content and/or composition within the hair follicles, that is, FHG and follicular hypotrophy, would be sufficient to disturb their anagen proliferative activity and force them to enter catagen. Given this, increasing the concentration of follicular proteoglycans and reactivating the dormant papillary cells provide a promising target for the prevention and treatment of TE as recently proposed by Thom [94]. Longitudinal studies with multiple scalp biopsies are needed to evaluate the correlation of clinical symptoms with the composition and concentration of proteoglycans in different parts of the hair follicles.

## 5. Proteoglycan-Based Therapies for Hair Loss

Although the structure and function of proteoglycans are becoming clearer, the science of using proteoglycans as therapeutic agents is still in its infancy. Currently, the only available and safe method is to administer an effective dosage of specific proteoglycans orally in the form of PRT. In practice, this unique approach has been explored in specific clinical areas such as rheumatology and skin aging. In a clinical trial on males and females with severe knee pain, oral salmon nasal proteoglycans have been proved to be effective in decreasing collagen degradation and promoting collagen synthesis [95]. As a therapy for skin aging, a marine-based proteoglycan complex significantly improved skin viscoelasticity and reduced the number of wrinkles and skin looseness in both men and women [96]. Nonetheless, the high potential of proteoglycans as intervention targets in neoplastic disorders [4] and hair growth disorders is still a largely uncharted frontier. It is quite dubious why a plethora of evidence pointing to the centrality of proteoglycans in the functionality of hair follicles [11, 13, 24, 97] has not yet encouraged the scientific community to explore feasible methods of modifying the follicular concentration or synthesis rate of proteoglycans.

Historically, the idea of treating hair loss by oral proteoglycans originated in the late 1970s as the fruit of a scientific endeavour by Jan Wadstein and Erling Thom. These researchers investigated the physiological links between the expression of specific proteoglycans and hair growth cycle and hypothesised that pharmacological application of these active macromolecules may yield a potential solution for hair loss while being desirably safe. This hypothesis was confirmed through a clinical study, conducted in 1991, in which a crude mixture of proteoglycans was being evaluated for another indication. During the trial, an accidental misplacement of an ultrasound scanning probe onto the higher temple with hair follicles rather than the facial skin has led to the discovery of visible changes in the structure of hair follicles induced by oral administration of proteoglycans [98]. Subsequent to this finding, good effort had been made in research centres in Denmark, Norway, and Sweden to formulate a specific enzymatic-extracted mixture of proteoglycans aimed to treat hair loss problems. The resulting product (Nourkrin® with Marilex®,Pharma Medico Aps, Aarhus, Denmark) has been found to produce promising results in different clinical settings [99–101], reviewed by Thom and colleagues [102]. As yet, this line of research has admittedly had limited effects on global standard therapeutic guidelines for hair loss management; however, it appears to be the time for reconsideration.

### 5.1. Oral PRT in Pattern Hair Loss (MPHL and FPHL)

Conventional treatment of PHL is mainly focused on minimising the stimulation of follicular androgen receptors by utilising antiandrogen medications, e.g., 5*α*-reductase inhibitors, as opposed to targeting the downstream mediators linking various environmental stimuli to the cellular dysfunction present in PHL-affected follicles. Approaching PHL beyond the androgen receptor is specifically essential in women since antiandrogen medications are not effective in FPHL [103]. As explained in Section 4.1, proteoglycans are important elements that modify several of the sex hormone receptor effectors, involved in hair growth. Thus, augmenting the concentration of follicular proteoglycans by intervention, e.g., PRT with Nourkrin® with Marilex®, may counteract the effects of sex hormones at a lower level than the receptor. This approach may terminate the vicious cycle of deficient ECM leading to cellular dysfunction that can end in PFA and miniaturisation. This theory explains the significant clinical efficacy of Nourkrin® in improving hair growth in both men and women with PHL (see below).

As described earlier, preliminary findings in the late 1970s heralded a line of clinical trials designed to evaluate the impact of an improved, proprietary formulation of enzymatic-extracted, marine proteoglycans rich in lecticans and decorin, marketed as Nourkrin® with Marilex®, on hair growth in both men and women. First, the clinical efficacy of Nourkrin® as a monotherapy was assessed in men and women with PHL through a six-month, randomised, placebo-controlled, clinical trial followed by an additional 6–12 months of open-label survey. Compared with the control group, PRT with Nourkrin®gradually increased hair count per unit area, which reached statistical significance after six months. This positive improvement resulted in considerably higher treatment satisfaction among treated individuals [100]. This study has subsequently been replicated in another group of individuals of both genders with PHL. In accordance with the previous trial, PRT with Nourkrin® has induced a significant increase of 35.7% in scalp hair count after 6 months, which showed strong correlation with subjective treatment satisfaction. Akin to the prior study, continuing PRT for a total of 12 months produced significantly improved patient satisfaction [101]. Building on these promising results, investigators have studied how Nourkrin® with Marilex® influences the quality of life in women with FPHL. 85 volunteers received treatment with Nourkrin® added to a number of topical cosmetic products. After six months of treatment, overall quality of life and its subcategories were significantly improved as measured by Kingsley Alopecia Profile [99].

Taken together, the published interventional evidence indicates that monotherapy or add-on treatment with a special blend of marine, enzymatic-extracted, proteoglycans (Nourkrin® with Marilex®) can reduce hair loss and enhance hair count in both men and women with PHL. It is of note that the first signs of clinical response have been spotted within 60 days of taking Nourkrin®, while both response rate and magnitude have improved by continuation of treatment for a total of 12 months. Plausibly, postponing catagen in active follicles and inducing anagen in dormant telogen follicles by this specific proteoglycan formula are responsible for the early clinical response. More gradual improvements in hair density and thinning (the tardive impact) are likely to be underpinned by a gradual augmentation in the synthesis ability of follicular cells during anagen. This effect minimises FHG and PFA giving an androgen-independent action in view of the fact that blocking androgens does not reverse hair thinning in PHL [85, 104]. It is also worth considering that none of the trials have reported posttreatment hair shedding with PRT with Nourkrin®, typical to medications such as minoxidil, which signifies a different mechanism of action. With no reports of significant treatment-related side effects in clinical trials, Nourkrin® with Marilex® is considered well tolerated and safe to use in hair loss patients.

### 5.2. Oral PRT in Telogen Effluvium (TE)

It is unfortunate to admit that faced with TE, the clinicians' repertoire is currently vacant and, there is no standard means available to effectively address the pathophysiology of TE. Depending on the type of TE, the following three pharmacodynamic targets have been suggested for a potential therapeutic intervention [90]:

1. Induction of anagen in telogen follicles
2. Inhibition of catagen initiation
3. Inhibition of exogen

In light of this, proteoglycans with their unique functional properties are obvious candidates to be part of a potential solution. Specific proteoglycans, such as decorin, versican, and perlecan, possess robust anagen-inducing activity [13] that has been verified in vivo [14, 24]. This unique effect can be harboured to promote anagen in TE-affected follicles with “delayed telogen release” and shorten telogen in postpartum women with “delayed anagen release” type of TE. Decorin has also shown capacity to inhibit catagen initiation [4] and prevent cellular apoptosis [39, 48], which predictably heralds catagen. These anagen-maintaining effects can potentially prevent stress- and drug-induced TE with “immediate anagen release” and be therapeutic in patients with “short anagen syndrome.” Thus, regulating abnormal anagen/telogen transition by PRT (Nourkrin® with Marilex®) imparts this approach its relevance as a therapy in various types of noninflammatory TE.

This targeted PRT may also prove to be of high value in the management of chronic TE, a difficult-to-manage condition defined as a diffuse scalp hair loss that persists for more than six months. Any of the Headington's functional types of TE can turn chronic with a frequently unspecified triggering event [105]. In approaching such a condition of undetermined pathogenesis, a versatile therapy, such as PRT, can be of high value.

From another perspective, the broad anti-inflammatory effects of follicular proteoglycans may further contribute to their anti-hair loss efficacy by subsiding the catagen-inducing effects of inflammatory cytokines and immune infiltration (Section 6.2). PRT with Nourkrin® may also be considered a type of “immune privilege restoration therapy” (Section 3.3). This intriguing prospect awaits confirmation by future randomised, controlled trials.

At this point, Nourkrin® with Marilex®, a specific blend of enzymatic extracted marine-derived proteoglycans, is the only available method of proteoglycan-based therapy for PHL with confirmed efficacy (Section 5.1). PRT with Nourkrin® is also a clear candidate for treating different types of TE, particularly postpartum TE, according to a solid theoretical support for efficacy and a desirable safety profile.

## 6. Pharmacology of Oral PRT

While enteral administration of proteoglycans has been proved to be effective in inducing biological changes in peripheral tissues, little is known about the pharmacological parameters of this novel class of nutraceuticals. Targeted ex vivo and in vivo studies are needed to better understand the absorption dynamics and action mechanisms of Marilex® that can lead to expanding its clinical application. Nonetheless, the available literature provides some insight into the pharmacokinetic and pharmacodynamic properties of oral proteoglycans and their degradation products.

### 6.1. Pharmacokinetics

Whether or not proteoglycan macromolecules and their moieties can be absorbed by the human intestine has long been a matter of debate. Among glycosaminoglycans, HA and CS are the most commonly prescribed forms and have been subjected to meticulous pharmacokinetic evaluations. Studies on rats and dogs have documented that ingested high-molecular weight HA can be absorbed and systematically distributed to the peripheral tissues [106]. We also know from in vitro studies that CS can survive the gastric and proximal intestinal degradation and reach the colon, where it is broken down to its disaccharide units by colonic microflora [107]. A smaller fraction of CS is absorbed intact along the small intestine by endocytosis and the rest as degradation products in the colon and the cecum [107]. Multiple in vivo studies in rats, dogs [108], horses [109], and humans [110, 111] have demonstrated that CS is bioavailable and can be quantified in systemic circulation after oral administration. It reaches its peak plasma concentration after 2 to 4 hours with an elimination half-life of around 6 hours [112, 113]. Evidence suggests that while molecular weight is inversely associated with absorption rate, multiple dosing considerably improves bioavailability [114]. Radioactive-labelling studies have shown a tropism of CS towards glycosaminoglycan-rich tissues such as the joint cartilage [108]. Nonetheless, these findings have not been completely undisputed [115] and need to be further established by larger studies.

The intestinal absorption dynamics of marine-based proteoglycans have been studied in rats using an everted-sac method. The findings verified that proteoglycans are absorbed effectively by jejunum and ileum in a dose-dependent manner, whereas absorption in the colon is dose independent but limited. Remarkably, the absorption level of proteoglycans was higher than CS in the jejunum and comparable in other parts of the bowel. Further exploration revealed that close to half of the proteoglycan absorption in the jejunum and the ileum is via clathrin-mediated endocytosis [116]. Furthermore, since bioactivity depends on sufficient bioavailability and bioaccessibility, positive outcomes produced by oral PRT with Nourkrin® in clinical trials in a variety of health conditions [95, 99–101, 117] may be taken as indirect evidence for oral bioavailability of specific proteoglycans. However, making reliable conclusions would require direct in vivo pharmacokinetic studies. Besides, the proportion of absorbed macromolecules over their degradation products and the share of each compound in inducing the overall pharmacological bioactivity are yet to be determined.

### 6.2. Mechanism of Action

Oral intake of Nourkrin® with Marilex® introduces a combination of bioactive proteoglycans to the gastrointestinal tract that may both affect the equilibrium of the gut and exert systemic effects. Since the pharmacokinetic data indicate that both intact proteoglycans and their degradation products are absorbed into the circulation, it may be assumed that they reach the hair follicles and amplify their proteoglycan content. This action can hypothetically be implemented by either or both of direct deposition and augmentation of cellular synthesis secondary to the increased input of xenobiotic building blocks. The resulting increase in the concentration of follicular proteoglycans improves FHG and prevents/delays progression towards PFA. This mechanism appears to, at least partly, explain the observed hair-stimulating and hair-thickening clinical efficacy of oral PRT with Nourkrin®. Although much further research is needed to illustrate a more complete picture, the followings are plausible molecular mechanisms through which PRT promotes the growth and survival of scalp hair follicles:

#### 6.2.1. Promoting Cell Proliferation and Survival

It is well evidenced that a critical concentration of ECM proteoglycans is required for inducing and maintaining the proliferation capacity of DP fibroblasts and follicular stem cells. In vitro research has demonstrated that marine-derived proteoglycans enhance the proliferation of human dermal fibroblasts through activation of Erk1/2 signalling pathway by their EGF-like module [118]. In parallel, marine proteoglycans have caused a prominent upregulation in both the proliferation and migration activity of fibroblasts. CS-containing side chains are proven to be indispensable for this bioactivity [119]. A more specific study has revealed that either enhanced endogenous expression or treatment with exogenous recombinant versican significantly stimulates fibroblast cell proliferation through the EGF-like motif of its G3 domain [120]. Later, this finding has been confirmed by showing that the versican level increases in aggregative DP cells, and its suppression by RNA interference can lead to a considerable loss of aggregative growth and proliferative ability of DP cells [34]. Further research has revealed that versican is the specific mediator of Wnt/*β*-catenin pathway in regulating the aggregative growth of DP cells [38].

The effect of externally administered animal-derived proteoglycans on stem cells has clearly been demonstrated by ex vivo experiments on human hematopoietic progenitor cells. Salmon cartilage proteoglycans affected the optimal binding affinity and kinetics of interactions between progenitor cells and early-acting cytokines and matrix components, which regulate the differentiation pathway of progenitor cells [121]. These lines of evidence denote that augmenting the concentration of follicular proteoglycans by intervention, e.g., PRT with Nourkrin®, can reasonably enhance the proliferation and survival of key follicular cell populations and thereby mitigate abnormalities in anagen initiation and maintenance.

#### 6.2.2. Immunoregulatory Properties

As stated in Section 4, perifollicular sterile inflammation and fibrosis are commonly found in all types of PHL with a viable possibility of having causative implications. In this context, the multifaceted bioactivity of proteoglycans and their glycan moieties may be harboured to control the alopecia-associated inflammatory reactions, specially that proteoglycans show direct and indirect (mediated by the intestinal microbiota, as elaborated later) involvement in the regulation of various stages of immune response [48, 63]. For instance, decorin inhibits the in vivo adhesion of polymorphonuclear leukocytes by downregulating the expression of intercellular adhesion molecules and syndecan-1 [122]. In another model, a marine-based proteoglycan complex effectively mitigated the progression of encephalomyelitis by suppressing T-helper cell lineages through the downregulation of interferon-*β*, *γ*, and pro-inflammatory interleukins and enhancing the expansion of regulatory T cells via FOXP3 proteins [123]. The anti-inflammatory efficacy of treatment with fish cartilage proteoglycans has further been verified in a model of obesity-induced inflammation. Throughout the eight weeks of intervention, proteoglycans provided a time-dependent mitigation of adipose tissue inflammation and suppressed the expression level of key pro-inflammatory mediators [124]. Although experimental evidence in humans is still lacking, it can sensibly be hypothesised that the anti-inflammatory properties of certain proteoglycans observed in a number of peripheral tissues can be extended to the hair follicles, and thus be considered for treating hair loss in humans.

#### 6.2.3. Antioxidative Properties

Decreased total antioxidant capacity due to oxidative stress has been observed in individuals with PHL [125] and is proposed to act as a pathogenetic factor in different types of hair loss. DP cells from PHL-prone follicles commonly exhibit increased levels of reactive oxygen species and reduced ability to handle oxidative stress, which leads to their premature senescence [126]. Conceivably, certain proteoglycans, such as versican, are involved in this pathology for being part of the cell's antioxidant defence system. In vitro, induced expression of versican provides protection from H_2_O_2_-induced apoptosis, and compared with controls, versican-expressing cells demonstrate superior adhesion and viability in response to oxidative stress [46]. Putatively, forming a pericellular barrier, inhibition of lipid (per)oxidation, scavenging superoxide anions, and mediating intracellular signal transduction pathways are among the antioxidant actions of proteoglycans [127]. This function is inarguably crucial for sustaining the viability of active follicular cells during anagen.

#### 6.2.4. Effects on Gut Microbiota

It is now known beyond doubt that the composition of gut microbiota is a determining factor in sustaining the normal physiology and immunological homeostasis of the human body. Hence, any modification in its balance can affect even the most distant tissues, such as the heart, brain, and skin.

The extent of support for a close connection between the gut and skin is sufficient to be formulated into a new, unifying model, namely, “the gut-brain-skin axis.” This model is based on the shared signals, cellular protagonists, and other similarities in the functions of nerve fibres and mast cells between the skin and intestine [128]. Moreover, there is strong evidence for the effectiveness of microbiome-modifying interventions in some skin disorders such as atopic dermatitis [129], cutaneous wounds [130], and hair growth disorders. With regard to the latter, studies in mice have demonstrated that probiotics may counteract the anagen-shortening effects of stress and reduce apoptosis in the hair matrix epithelium [131]. On the contrary, intestinal dysbiosis is involved in the pathophysiology of alopecia [132].

Evidently, a viable way of modifying the gut microbiota is by shifting the nutrients on which the gut bacteriae thrive, primarily glycans (prebiotics). Therefore, the long-term administration of specific glycan-rich marine-based proteoglycans has the potential to serve as a prebiotic complex. This notion has been verified by in vivo experiments on C57BL/6 mice, where salmon cartilage proteoglycans have caused a significant increase in the population of saccharolytic bacteriae, which produce short-chain fatty acids (SCFAs) from complex glycans [133]. This class of fatty acids are effective anti-inflammatory agents that protect the intestinal barrier and regulate the immune system in humans [134]. It is thus sensible to speculate that some aspects of the effects of oral proteoglycan-based therapies on follicular and skin disorders may be mediated by their boosting effect on the production of SCFAs. Specially in the case of alopecia areata, SCFAs contribute to resolving local inflammation around the hair follicles [135]. However, interventional clinical studies are required to elucidate this potential pharmacological effect of oral PRT, which will open up new prospects for therapeutic intervention in various hair growth disorders.

## 7. Summary and Conclusion

We argued in this review that while the structural significance of proteoglycans is old knowledge, their fundamental functional roles in various organs, including the hair follicle, still continue to surface, thanks to the development of novel investigation techniques. Clear evidence shows that specific proteoglycans are present in various parts of the hair follicle with a sharp contrast to the surrounding dermal zones. It appears that this unique pattern of distribution is proactively determined by the functional role of each proteoglycan. During telogen-to-anagen transitions, the expression of a number of proteoglycans, particularly in the mesenchymal parts of the follicle, undergoes radical changes, which exhibits a perfect association with the activity of proliferating cell populations. This targeted redistribution, in line with emerging evidence from mechanistic research, suggests that proteoglycans contribute to maintaining and regulating the hair growth cycle. Specific proteoglycans support DP fibroblasts in inducing anagen, enable follicles to sustain anagen, and contribute to follicular immune privilege.

Given their broad bioactivity, the altered dynamics of proteoglycan metabolism can potentially cause a range of abnormalities in growth and cycling of the hair follicle. Controlled observations are indicative of a decay in the proteoglycan content of the mesenchymal follicular components in PHL-affected follicles. A state of FHG may accordingly represent a common denominator between the pathogenesis of common forms of hair loss caused by the dysregulation of hair growth cycle, i.e., PHL and TE. In PHL-prone follicles, hormonal signals induce a catabolic state, predominantly in the DP, that leads to progressive levels of FHG. A low per-cell concentration of key proteoglycans enhances the impact of downstream effectors of the androgen receptor and thereby accelerates the degradation process of hair follicles, known as PFA. Advanced PFA presents itself clinically as follicular miniaturisation. The involvement of specific proteoglycans in the pathophysiology of TE relies on the integral function of these bioactive macromolecules in regulating the cycling behaviour of the hair follicle. Accordingly, a disturbed anabolism of proteoglycans, e.g., secondary to oestrogen withdrawal, would disrupt their anagen-sustaining influence and leads to an increase in telogen/anagen ratio in scalp follicles. This modern understanding of the centrality of proteoglycans in the pathogenesis of hair growth disorders has paved the way for the development of a proteoglycan-based therapy (Nourkrin® with Marilex®).

Currently, the only available form of proteoglycan-based therapy aimed for treating common hair growth disorders, MPHL, FPHL, and TE, is a combination of specific enzymatic-extracted marine-derived proteoglycans rich in lecticans and small leucine-rich proteoglycans administered by mouth (Marilex® ). The safety and efficacy of this form of treatment have been examined in several clinical studies in both men and women with PHL. According to the published reports, first signs of efficacy were detectable after 60 days of treatment (immediate impact). However, continuing PRT with Nourkrin® for a total of 6 to 12 months has yielded significant objective improvements in scalp hair density, rate of hair growth, and spontaneous hair loss (tardive impact), which resulted in significantly higher treatment satisfaction and enhanced quality of life than control subjects. Nourkrin® was well tolerated and caused no considerable side effects. With regard to TE, proteoglycans with anagen-inducing and catagen-delaying effects can theoretically serve as preventive or therapeutic compounds in all forms of noninflammatory TEs, especially in the common postpartum TE.

With respect to the action mechanisms of PRT with Nourkrin®, promoting cell proliferation and survival of fibroblasts and stem cells via growth factor-like domains of bioactive proteoglycans together with their immunoregulatory and antioxidative properties and a positive impact on gut microbiota have been proposed. In vivo pharmacokinetic assessments have indicated an adequate gastrointestinal bioavailability for marine-based proteoglycans that enables oral PRT to affect peripheral organs such as the hair follicles. Proteoglycans and certain glycosaminoglycans are absorbed in their intact forms via endocytosis mainly in the small intestine, a process that can be enhanced by multiple dosing.

Overall, the aggregation of evidence leads us to believe that specific proteoglycans are neglected key players in pathophysiology of hair growth disorders, which deserve more clinical and research attention. We have tried in this review to highlight the extensive roles of proteoglycans in the normal functioning of the hair follicle to explain the significance of their dysmetabolism in the pathogenesis of common hair growth disorders. In doing so, we have introduced the concepts of FHG and PFA as useful tools to understand a phenomenon that appears to happen in various forms of hair loss. FHG and PFA are clinically presented with a disrupted hair growth cycle and/or follicular miniaturisation. Unambiguously, the efficacy of oral PRT with Marilex® in clinical studies further supports the involvement of FHG and PFA in the pathogenesis of PHL. In light of this, we believe that depending on the clinical situation, oral PRT with Nourkrin® can be viewed as a versatile monotherapy or an effective add-on treatment to improve the effectiveness of traditional hair loss medications via a completely different mode of action.

Scientists have just started to explore the nature and potential of PRT with a specific enzymatic-extracted marine-based proteoglycan formulation (Marilex®). As of today, the following research topics are among the most curious subjects around PRT that await further experimental clarification: (A) the impact of specific proteoglycans on anagen-inducing and anagen-suppressing pathways in follicular fibroblasts and stem cells, (B) correlation between the concentration of follicular proteoglycans and the stage of PHL and TE, (C) alterations in the follicular expression of key proteoglycans after PRT with Nourkrin® with Marilex®, and (D) pharmacokinetic assessment of oral administration of proteoglycans.
